# Intracellular Targeting of CEA Results in Th1-Type Antibody Responses Following Intradermal Genetic Vaccination by a Needle-Free Jet Injection Device

**DOI:** 10.1100/tsw.2007.138

**Published:** 2007-06-12

**Authors:** Susanne Johansson, Monica Ek, Britta Wahren, Richard Stout, Margaret A. Liu, Kristian Hallermalm

**Affiliations:** ^1^ Department of Microbiology and Tumor and Cell Biology, Karolinska Institutet, Stockholm, Sweden; ^2^ Swedish Institute for Infectious Disease Control, Stockholm, Sweden; ^3^ Center for Molecular Medicine, Karolinska Institutet, Stockholm, Sweden; ^4^ Bioject Medical Technologies Inc., Tualatin, OR, USA

**Keywords:** carcinoembryonic antigen, genetic vaccination, antibody response, Biojector

## Abstract

The route and method of immunization, as well as the cellular localization of the antigen, can influence the generation of an immune response. In general, intramuscular immunization results in Th1 responses, whereas intradermal delivery of DNA by gene gun immunization often results in more Th2 responses. Here we investigate how altering the cellular localization of the tumor antigen CEA (carcinoembryonic antigen) affects the quality and amplitude of DNA vaccine-induced antibody responses in mice following intradermal delivery of DNA by a needle-free jet injection device (Biojector). CEA was expressed either in a membrane-bound form (wild-type CEA) or in two truncated forms (CEA6 and CEA66) with cytoplasmic localization, where CEA66 was fused to a promiscuous T-helper epitope from tetanus toxin. Repeated intradermal immunization of BALB/c mice with DNA encoding wild-type CEA produced high antibody titers of a mixed IgG1/IgG2a ratio. In contrast, utilizing the DNA construct that resulted in intracellular targeting of CEA led to a reduced capacity to induce CEA-specific antibodies, but instead induced a Th1-biased immune response.

## INTRODUCTION

The route and method of DNA delivery can impact the outcome of vaccination with gene-based constructs. By targeting the same antigen to different tissues by intramuscular injection or biolistic (gene gun) intradermal immunization, the resulting immune response can be of either a predominantly Th1 or a Th2 type[1], favoring either the cellular or humoral arm of the immune system. In addition to the route and method of delivery, the nature of the induced immune response is also influenced by the localization of the expressed antigen. Several investigations into how cellular localization of the antigen affects the generation of an immune response after regular intramuscular[2,3,4,5,6,7,8] or intradermal gene gun[9]

*Contributed equally. **Corresponding author.
©2007 with author.
Published by TheScientificWorld; www.thescientificworld.com immunization have been reported. A study on intramuscular injection of different forms of the model antigen ovalbumin (OVA) showed that secreted OVA induced higher antibody levels than a membrane-bound or cytoplasmic form of the antigen. While immunization with a gene encoding a secreted form of OVA led to a Th2-biased immune response, immunization with genes encoding membrane-bound and cytoplasmic OVA resulted in a Th1-type response[2]. Similarly, intradermal gene gun immunization of cytoplasmic OVA resulted in a Th1-type response, while the secreted or membrane bound forms of OVA induced a mixed response[9].

While gene gun immunization is carried out by propelling DNA-coated gold particles directly into the cells of the skin, the needle-free jet injection device, Biojector, delivers DNA as a solution by creating an ultrafine stream of high-pressure fluid that penetrates the skin, and is distributed in the dermis or intramuscularly depending on the amount of pressure used. Although the location of administration is the same, the actual delivery or uptake of DNA into the expressing cells might be entirely different. Therefore, it is possible that the influence of the localization of the expressed antigen on immune stimulation could differ between the two methods.

For cancer vaccination, the generation of a robust Th1-type immune response is usually considered preferable. Th1 cells produce proinflammatory cytokines like IFNγ and TNFα, which support the stimulation of tumor-specific CD8+ T cells with cytotoxic capacity. Moreover, the Th1 milieu shifts the balance of the humoral immune response towards production of antibodies of IgG1 and IgG3 subclasses in humans[10] and IgG2a in mice[11,12], which are important for humoral effector functions, such as complement lysis and antibody-dependent cellular cytotoxicity (ADCC)[13]. Therefore, knowledge about how antigen characteristics, immunization method, and the route of delivery influences the generation of an antitumor response is of importance.

We have previously had good experience using the Biojector for intradermal delivery of HIV DNA vaccines in both mice and humans[14,15,16], and plan on employing this device for cancer vaccination of human subjects in the future. Here we investigate how cellular antigen localization affects the quality and amplitude of CEA DNA vaccine-induced antibody responses following intradermal Biojector immunization.

## MATERIALS AND METHODS

### Plasmids and Antibodies

The three CEA-containing plasmid constructs — p91023(B), pKCEA6, and pKCEA66 — were previously described[17]. Briefly, p91023(B) contains the full-length, wild-type CEA sequence driven by the adenovirus major late promoter and with an SV40 poly A tail[18]. The pKCEA6 encodes a truncated form of wtCEA in which the N- and C-terminal signal sequences were removed[17], and the remaining part of the CEA gene was inserted into the pKCMV vector. This vector holds a CMV promoter, a HPV16 poly(A) signal, and an *Escherichia coli* origin of replication and encodes kanamycin resistance. In pKCEA66, the truncated gene was fused to the promiscuous helper T-cell epitope QYIKANSKFIGITEL (representing amino acids 830–844 of tetanus toxoid)[17]. Mouse monoclonal antibodies directed against human CEA used were clone II-7 (DakoCytomation Norden AB, Stockholm, Sweden), Col-1 (BD Pharmingen, Franklin Lakes, NJ), 1C11 (Abcam, Cambridge, U.K.). The rabbit antihuman CEA ab15987 was from Abcam Ltd (Cambridge, U.K.). TRITC-labeled goat antimouse IgG and FITC-labeled swine antirabbit IgG were obtained from DakoCytomation (Stockholm, Sweden). HRP-conjugated rabbit antimouse IgG and HRP-conjugated goat antimouse were from DakoCytomation (Stockholm, Sweden). The HRP-conjugated goat antihuman antibody was purchased from Bio-Rad Laboratories, (Richmond, CA) and the HRP-conjugated rabbit antigoat antibody was from DakoCytomation (Stockholm, Sweden).

## Cell Lines

HeLa and HEK293 cells were propagated in Dulbecco's modified Eagles medium (DMEM) supplemented with 10% heat inactivated fetal calf serum (Sigma), 100 IU/ml of penicillin, 100 μg/ml streptomycin, and 2 m*M* L-glutamine (Sigma-Aldrich Sweden AB, Stockholm, Sweden).

## Western Blot Analysis of CEA Expression

HeLa or HEK293 cells were transiently transfected using Lipofectamine 2000 (Invitrogen AB, Stockholm, Sweden) according to the manufacturer's recommendation. After 48 h, transfected cells were detached by trypsinization. After washing in PBS, the cells were lysed in Laemmli buffer (Bio-Rad, Hercules, CA). Gel electrophoresis and immunoblotting were performed using the Readygel Electrophoresis System (Bio-Rad, Hercules, CA).

## Immunofluorescence

Cells growing on glass cover slips were transiently transfected with CEA using Lipofectamine 2000. The cells were fixed in ice-cold acetone/methanol (80:20) for 10 min at +4°C. After fixation, the cover slips were kept at room temperature for 30 min, washed two times with PBS, and incubated with CEA-specific antibodies (mixture of mouse monoclonals clone II-7 1:10, Col-1 1:15, and 1C11 1:15 or rabbit anti-CEA IgG 1:200) in PBS for 30 min at room temperature. After washing to remove unbound antibody, specific staining was visualized by incubation with TRITC-labeled goat antimouse IgG or FITC-labeled swine antirabbit antibodies. Following additional washing in PBS and water, slides were mounted in PBS/glycerol (1:9) and kept in the dark at +4°C until analysis. Surface staining for CEA was performed on non-permeabilized HeLa cells using the Col-1 monoclonal (1:10 dilution). After staining the cells were fixed in 4% paraformaldehyde.

## CEA Quantification in Growth Medium

Growth medium from HeLa cell cultures, transiently transfected with the different CEA constructs, was collected 72 h post-transfection, centrifuged to remove potential residual cellular debris, and stored at –20°C for subsequent quantification of soluble CEA content. Culture supernatants from transfected cells were concentrated using Centricon centrifugal filter devices Ultracel YM-50 (Millipore, Billerica, MA) with a molecular cut-off of 50 kDa, according to the manufacturer's recommendation.

The CEA protein content in growth medium from transfected cells was quantified by a CEA inhibition ELISA as described previously[17]. Briefly, rCEA-coated plates were blocked with 5% milk in PBS for 2 h at 37°C. Concentrated growth medium from transfected cells and recombinant CEA protein was serially diluted in 2.5% milk in PBS and incubated for 1 h at 37°C with monkey antihuman CEA sera at a final dilution of 1/6000. Growth medium from the CEA expressing human tumor cell line LS174T was used as a positive control. Plates were washed with ELISA buffer (0.05% Tween20, 0.15 *M* NaCl in $dH_2O$) and samples preincubated with monkey sera were added to the plates. After 2-h incubation at 37°C, plates were washed and goat antihuman HRP conjugated IgG diluted 1/3000 in 1.25% milk in PBS was added. Two hours later, the plates were developed by addition of O-phenylene diamine buffer. The reaction was stopped by addition of 2.5 *M* $H_2SO_4$, and the absorbance was measured at 490–650 nm. The amount of CEA protein present in growth medium was calculated from a titration curve obtained by serial dilution of rCEA. Expression of the transgenes was verified by Western blot analysis of transfected cell lysates.

### Immunizations

BALB/c mice (eight in each group) were given three monthly intradermal immunizations of 200-μg plasmid DNA (wtCEA, CEA6, CEA66, or a pKCMV control plasmid) by Biojector 2000 (Bioject Medical Technology Inc., Tualatin, OR).

### CEA ELISA

Plates were coated with 0.1 μg per well of rCEA (Protein Science, Meriden, USA) diluted in 0.05 *M* $Na_2CO_3$ (pH 9,6) and incubated at room temperature over night. After washing in ELISA buffer (0.05% Tween20, 0.15 *M* NaCl in $dH_2O$), the plates were blocked in 5% milk in PBS for 2 h. Sera from immunized mice diluted in 2.5% milk in PBS were added to the plates, and following incubation over night at 37°C, excess sera was removed by washing in ELISA buffer. After a 2-h incubation with a goat antimouse HRP conjugate diluted 1:4000 in 1.25% milk to detect CEA-specific antibodies, the plates were washed with ELISA buffer and developed by addition of O-phenylene diamine buffer (Sigma) activated with $H_2O_2$. After 10 min, the reaction was stopped by addition of 2.5 *M* $H_2SO_4$ and the optical density was read at 490 and 650 nm. A monoclonal mouse anti-CEA Ab (1C11) (AbCam, Cambridge, U.K.) diluted 1/100 was used as a positive control. For IgG1/IgG2a subclass determination, CEA-specific antibodies were detected using goat antimouse IgG1 or IgG2a antibodies at 1:5000 concentration (DAKO Sweden AB, Stockholm, Sweden) followed by an HRP-conjugated rabbit antigoat antibody diluted 1:4000.

## RESULTS

### Expression of the CEA Plasmid Constructs in Human Cells

Mammalian expression of the CEA DNA vaccine constructs was analyzed by Western blot on transiently transfected HEK293 (Fig. 1) or HeLa (data not shown) cells. All three proteins (wtCEA, CEA6, CEA66) were readily detected at 48 h post-transfection (Fig. 1). Wild-type CEA has a molecular weight of about 180–200 kDa, and a band of the corresponding size was detected in total cell lysates from p91023(B)-transfected cells (Fig. 1) matching the size of CEA detected in a lysate of LS174T cells, a cell line expressing endogenous CEA (Fig. 1). Cells transfected with the wild-type construct also gave rise to a band of around 150 kDa, matching that of an early, not yet fully glycosylated, form of CEA[19]. In contrast, detection of CEA in samples from cells expressing the truncated CEA proteins CEA6 and CEA66 (lacking signal peptides) revealed a band of about 80 kDa, corresponding to the reported size of nonglycosylated CEA (78 kDa)[20].

### Removal of the CEA Signal Peptides Alters Protein Localization and Prevents Secretion of CEA from Human Cells

In order to understand fully how the cellular localization of the antigen affects immunization outcome following intradermal Biojector administration of the vaccine, we studied localization of the different CEA protein products in detail. Anticipating that removal of the N-terminal signal sequence of CEA would prohibit cotranslational translocation of the CEA protein into the endoplasmic reticulum, thereby prevent transport of the truncated CEA proteins to the cell surface, we performed immunofluorescent staining of CEA-expressing HeLa cells to analyze the effects of signal sequence deletion on the cellular distribution of CEA. As shown in Fig. 2, wild-type CEA, which is attached to the plasma membrane by a GPI-anchor[21,22], accumulated

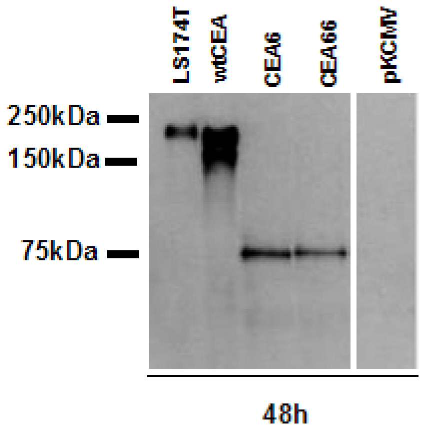

**FIGURE 1.** Expression of the CEA constructs**.** HEK 293 cells were transiently transfected with the different CEA constructs. At 48 h post-transfection, the cells were harvested and samples analyzed by western blot. CEA-specific bands were detected by chemiluminescence using a mouse antihuman CEA (DAKO clone II-7), as primary antibody.

around the edges of the cells, a clear sign of membrane localization. In contrast, the truncated CEA proteins CEA6 and CEA66 were distributed throughout the cytoplasm and showed no signs of increased staining at the cell boundaries. Interestingly, cells expressing the CEA6 construct often displayed perinuclear aggregation of CEA protein (Fig. 2B), a phenomenon that was not as common in cells expressing the CEA66 construct containing the Tet-epitope. Since the normal site of expression of wild-type CEA is on the outside of the plasma membrane[18,21,22], we also performed immunofluorescent staining of nonpermeabilized cells to verify and more accurately distinguish between plasma membrane localization of the wild-type protein and the intracellular staining pattern of the truncated constructs. As shown in Fig. 3, no CEA protein could be detected on the surface of cells expressing either CEA6 or CEA66 constructs, as assessed by fluorescence microscopy. However, cells expressing the wild-type form of CEA displayed a homogenous expression of CEA covering the entire cell surface.

CEA is shed from the surface of both normal and cancerous cells of the colon via a nonproteolytic mechanism mediated by a phospholipase[22,23,24]. Since the production of soluble CEA protein products could have an effect on what type of immune responses are induced by the modified CEA vaccine constructs, we investigated whether the truncated forms of CEA also could be secreted from the expressing cell. As shown in Fig. 4, culture supernatant from HeLa cells transfected with wtCEA or the LS174T cell line expressing endogenous CEA contained similar amounts of soluble CEA. In contrast, cells expressing the truncated CEA6 or CEA66 constructs did not secrete detectable levels of CEA (Fig. 4), arguing that the truncated CEA protein products indeed are retained inside the cell.

## Cytoplasmic Antigen Localization Following Intradermal Biojector Immunization Results in Reduced Antibody Responses in BALB/c Mice, but Shifts Antibody Responses Towards a Th1 Profile

To investigate how intracellular targeting of CEA would impact the induction of CEA-specific antibody responses after intradermal Biojector vaccination, BALB/c mice were immunized three times at a 4-week interval, using the different CEA constructs. At 14 days after the third immunization, serum from mice immunized with a construct encoding wild-type CEA contained extremely high titers of CEA-specific

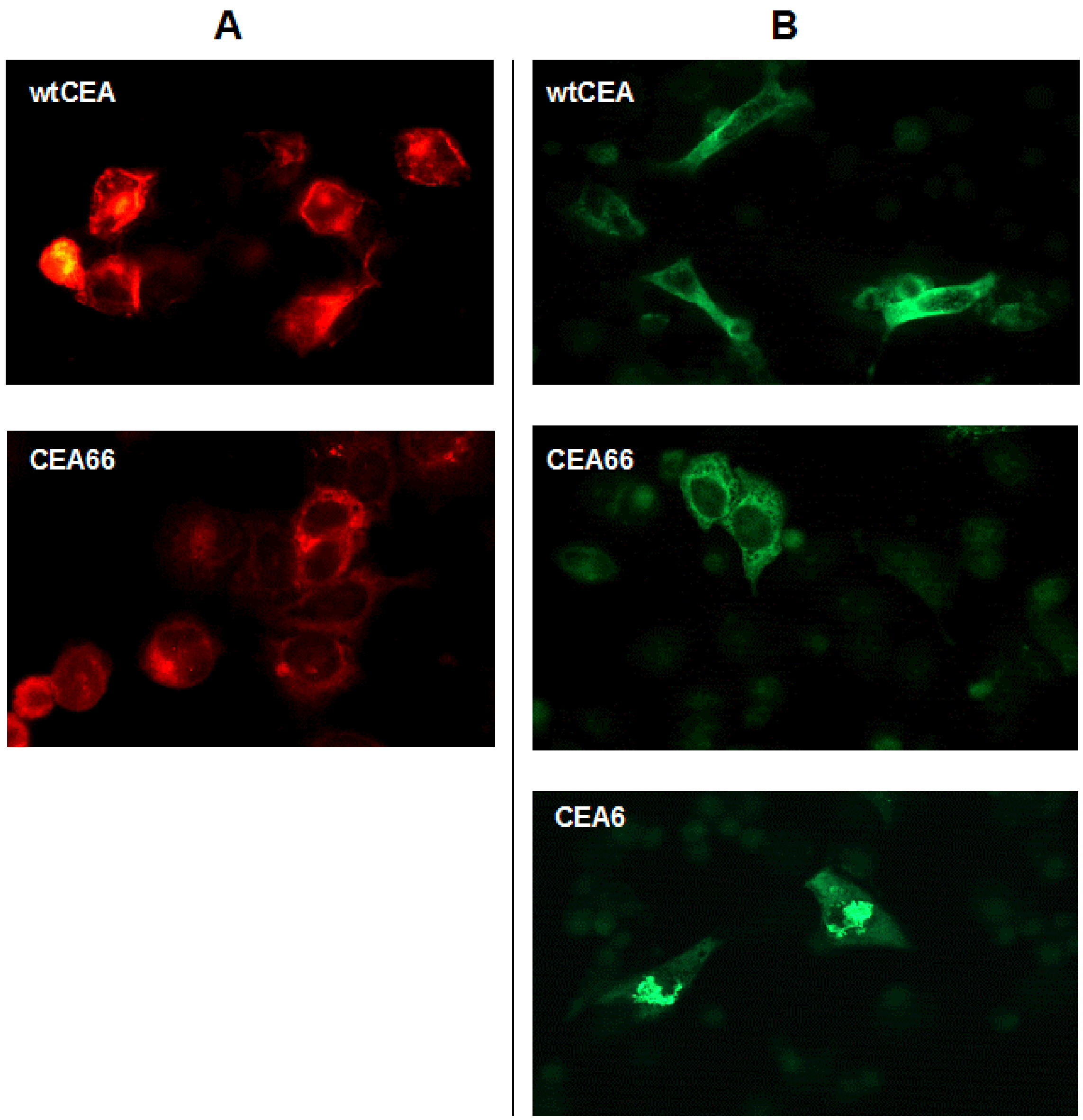

**FIGURE 2.** Deletion of signal peptides alters the cellular localization of CEA**.** HeLa cells, transiently transfected with different CEA constructs, were fixed and permeabilized in acetone:methanol (80:20%), and stained with: (A) a mixture of mouse monoclonal antibodies (DAKO clone II-7 1:10, Col-1 1:15, and 1C11 1:15) and visualized with TRITC-labeled goat antimouse IgG; (B) rabbit antihuman CEA followed by a FITC-labeled swine antirabbit antibody.

antibodies with an endpoint titer of $10^6$ (Fig. 5). Immunization with CEA6 DNA still resulted in high CEA-specific antibody titers of $10^4$ (Fig. 5), however, repeated intradermal administration of plasmid DNA encoding CEA66 (CEA6 fused to a tetanus T-helper epitope) did not raise the level of antigen-specific antibodies above background (Fig. 5, data not shown).

We also performed IgG2a/IgG1 subclass analysis of the induced CEA-specific antibody responses to estimate the induced T-helper profile. While we considered the antibody response to CEA66 to be too low for subclass analysis, repeated intradermal immunization with wild-type CEA led to a robust and balanced immune response, with an IgG2a/IgG1 ratio of approximately 1 (Fig. 6A,C). In contrast, the IgG2a/IgG1 ratio in CEA6-induced responses was around 2, indicating a shift towards a Th1 type of immune response (Fig. 6B,C).

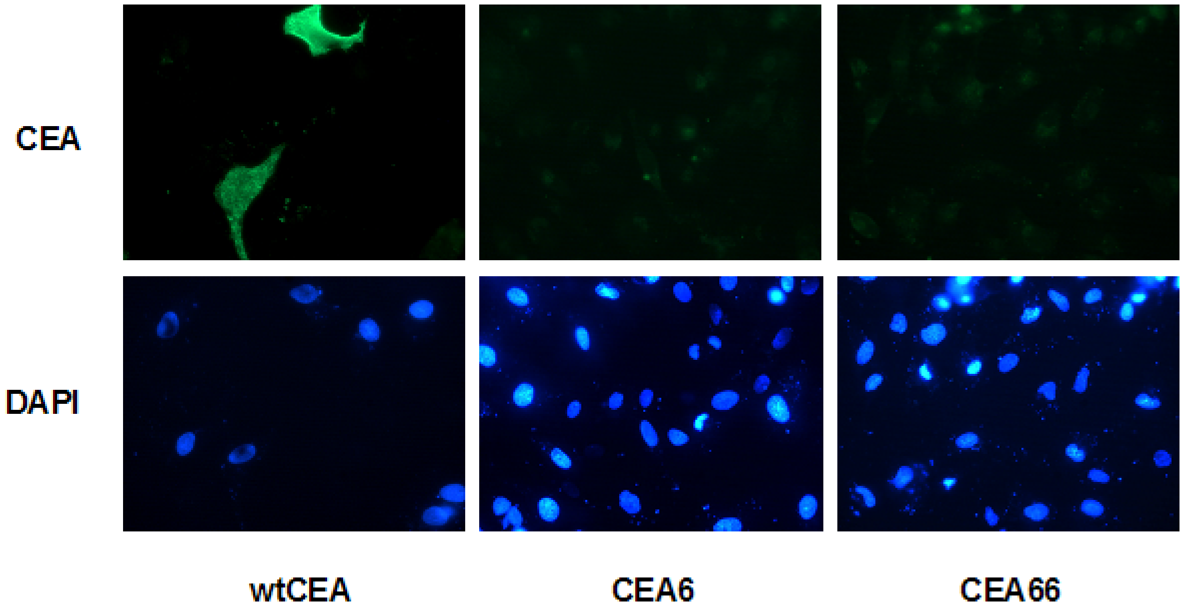

**FIGURE 3.** Signal sequence deletion prevents CEA transport to the cell surface. HeLa cells were transfected with wt CEA p91023(B), pKCEA6, or pKCEA66, and stained with rabbit antihuman CEA 48 h post-transfection. Counterstaining with DAPI was performed to indicate cell nuclei.

## DISCUSSION

To investigate how cellular antigen localization influences immune induction following intradermal needle-free jet injection, we have employed three CEA-encoding DNA constructs: wtCEA, CEA6, and CEA66. The constructs were compared with respect to the expression pattern and cellular localization of the protein products in mammalian cells, and investigated in relation to their capacity to induce CEA-specific antibody responses in mice.

All three constructs could be readily expressed in human HeLa (Fig. 4B) and HEK293 cells (Fig. 1). The detection signal from cells expressing the wild-type CEA was always stronger than that obtained with the constructs encoding the truncated CEA proteins. There can be several explanations for this. Differences in promoter strength between the adenovirus major late promoter controlling wtCEA expression from the p91023(B) plasmid and the CMV promoter controlling expression of the truncated CEA6 and CEA66 constructs from the pKCMV vector is a possible, however unlikely, explanation. A study comparing the activity of several promoters, including the CMV promoter and the adenovirus 2 major late promoter, demonstrated that the CMV promoter was superior to all other promoters examined[25]. Another explanation could be that the antibody (clone II-7) used by us to detect CEA expression in western blot has a higher affinity for the glycosylated wild-type CEA than the nonglycosylated truncated forms of CEA. Indeed, analysis of the epitope specificity of this antibody suggested that antibody binding occurs to a conformation-dependent epitope involving carbohydrate structures[26], which presumably would be absent on the nonglycosylated truncated forms of CEA (CEA6, CEA66). A third explanation could be differences in subcellular localization and degradation of the CEA protein products. Intracellular proteins like CEA6 and CEA66 should be more accessible for proteasomal degradation than the wtCEA, an extracellular membrane protein, which could result in a lower net amount of whole protein in the cell during steady-state conditions[27].

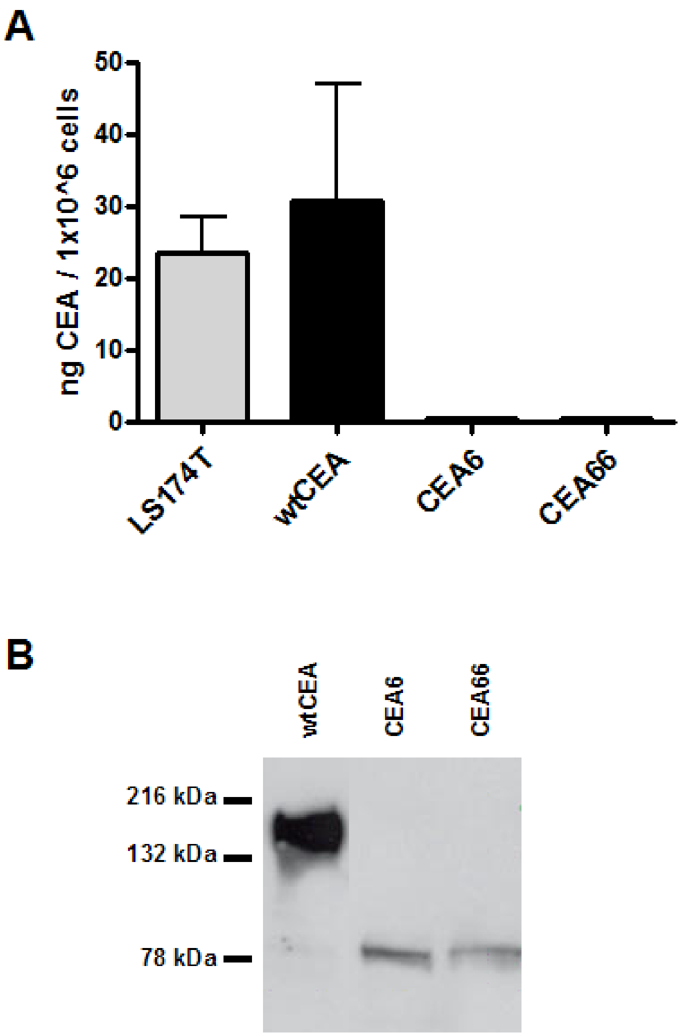

**FIGURE 4.** Truncated CEA proteins are retained within the producing cell. (A) Concentrated culture supernatants from CEA-transfected HeLa cells were subjected to an CEA inhibition ELISA. The amount of soluble CEA in the culture medium was expressed as ng CEA per 1 million cells. The mean ± SEM of three experiments is shown in the figure. (B) A representative western blot showing CEA expression by transfected HeLa cells used in panel A.

The sequence of wild-type CEA contains both N- and C-terminal signal peptides. The N-terminal signal peptide ensures proper cotranslational translocation of the wild-type CEA polypeptide into the ER, where it is heavily glycosylated, and its subsequent transport to the plasma membrane[19]. During post-translational processing of the protein, the C-terminal signal sequence is removed and replaced with a glycosylphosphatidylinositol (GPI) membrane anchor[21,28]. Indeed, intracellular immunofluorescent staining of cells expressing the wild-type CEA construct revealed a staining pattern typical of a membrane protein, with intensified staining around the edges of the cell, indicating accumulation of the CEA protein at the plasma membrane (Fig. 2), a finding that was confirmed by immunofluorescent staining of surface CEA on nonpermeabilized cells (Fig. 3).

Our modifications of the CEA genes in the CEA6 and the CEA66 constructs, which include the removal of both signal peptides, should prevent the transfer of CEA polypeptides into the endoplasmic reticulum and result in the production of nonglycosylated protein products, which are retained in the cytoplasm. Cells expressing the truncated CEA proteins (CEA6, CEA66) displayed a more even cytoplasmic staining pattern expected of an intracellular protein (Fig. 2), but did not express CEA at the plasma membrane (Fig. 3). In detail, CEA6-expressing cells (lacking both signal peptides) often exhibited perinuclear aggregation of CEA6 protein (Fig. 2B), perhaps as a consequence of defective intracellular transport. Intracellular staining for the CEA66 protein also revealed the appearance of cytoplasmic vacuoles from which CEA66 protein was excluded (Fig. 2), a phenomenon that may be associated with the function of the translocation domain of tetanus toxin, from which the epitope is derived[29].

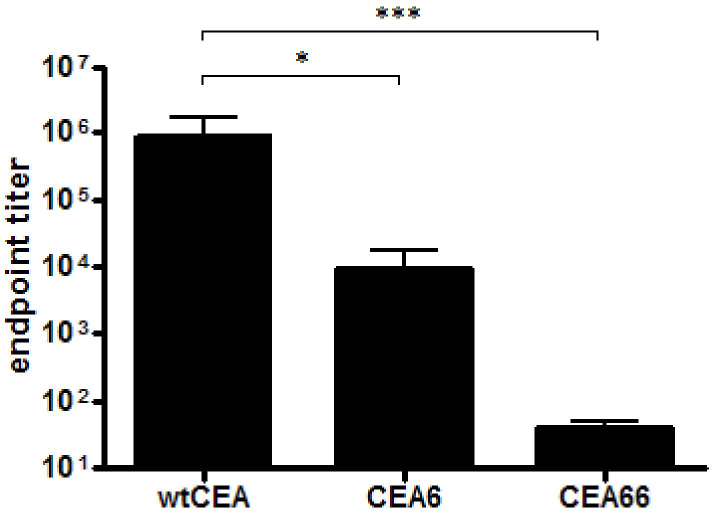

**FIGURE 5.** Intracellular targeting of CEA results in reduced CEA-specific antibody responses after intradermal Biojector immunization. Serum was taken 2 weeks after the third DNA immunization and subjected to a CEA ELISA. Bars show the mean endpoint titer of each group of immunized animals. * and *** indicate a statistically significant difference of $p < 0.05$ and $p < 0.001$, respectively.

That the truncated CEA protein products indeed are retained within the producing cell was confirmed by performing a CEA inhibition ELISA on concentrated culture supernatants from cells expressing the different CEA constructs. Supernatant from HeLa cells expressing wild-type CEA contained similar levels of soluble CEA protein, as did supernatants from the LS174T cell line expressing endogenous CEA (Fig. 4). In contrast, no soluble CEA could be detected in supernatants from cells expressing the CEA6 or CEA66 constructs, demonstrating that truncated CEA proteins are retained within the expressing cell.

The cellular localization of the CEA antigen did have an impact on the resulting immune responses. As expected, immunization with the wtCEA construct, resulting in production of both membrane-bound and soluble CEA, generated antibody titers of high magnitude (Fig. 5). This is in agreement with previous studies showing that secreted antigens are more efficient in inducing antibody response, both after intramuscular and intradermal gene gun immunizations[2,7,8,9]. On the other hand, intracellular targeting of CEA through deletion of the CEA signal sequences resulted in a decreased, but still potent, antibody response (Fig. 5). The decreased capacity of cytoplasmic CEA to stimulate a humoral response could be a result of increased intracellular degradation of the protein product, as suggested by the Western blot results (Fig. 1). This would reduce the availability of native protein for efficient antibody induction[30,31,32]. Interestingly, fusion of a promiscuous tetanus T-helper epitope to the truncated CEA construct (CEA66) did not result in increased antibody production, but rather had a negative impact on the induction of antibody responses to CEA (Fig. 5). It is possible that the addition of this epitope, stemming from a protein translocation domain, further prohibited release of whole CEA66 protein for B-cell receptor recognition. However, the negligible antibody response observed after repeated immunization with CEA66 DNA could be readily boosted by immunization with recombinant CEA

protein, demonstrating the antibody priming capacity of CEA66 DNA. Previously, tetanus T-helper epitopes in DNA vaccines have mostly been used in combination with secreted or soluble antigens[33,34,35].

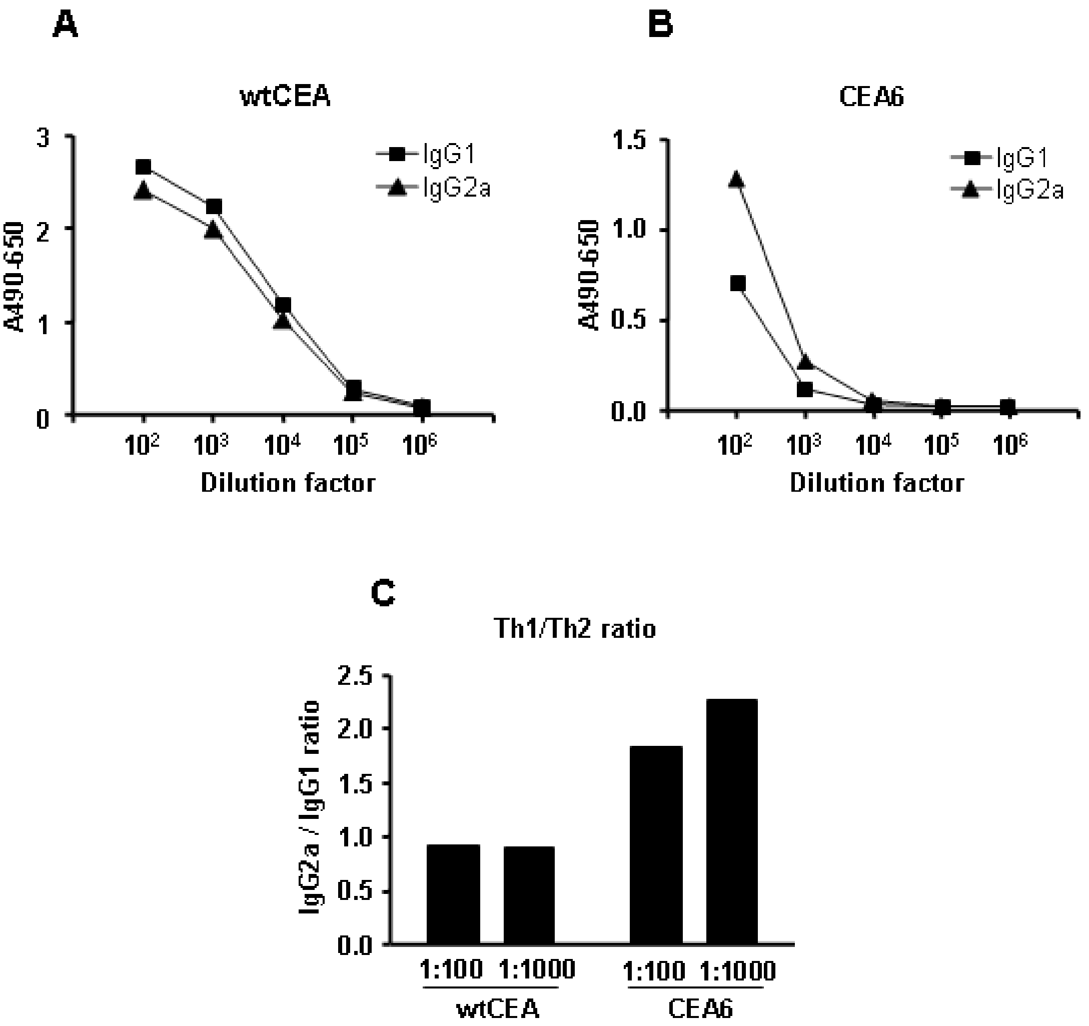

**FIGURE 6.** Cytoplasmic antigen localization tilts the antibody response to CEA towards a Th1 profile. Three weeks after the third immunization, serum was collected and pooled before analyzed for CEA-specific IgG1 and IgG2a antibodies. The levels of CEA-specific antibodies of IgG1 and IgG2a subclasses from animals immunized with wtCEA (A) and CEA6 (B) were determined. The IgG2a/IgG1 subclass ratio at serum dilutions of 1:100 and 1:1000 are shown in panel C. One representative experiment out of two performed is shown in the figure.

The main source of antigen for presentation on MHC class II molecules, which are recognized by CD4+ T-helper cells, derives from internalized extracellular proteins. Therefore, it is possible that conjugation of the Tet epitope to a nonsecreted antigen does not allow for optimal induction of T-cell help since the amount of exogenous antigen available for uptake by antigen-presenting cells will be limited.

The ultimate goal of cancer vaccination is the induction of cytotoxic T cells that can effectively target and lyse antigen-presenting tumor cells. Therefore, a tumor DNA vaccine should optimally lead to the induction of a Th1 type of immune response, which is generally considered to drive cellular immune responses[36]. A Th1-type immune response is associated with production of cytokines like IL-2 and IFNγ[36], but also affects what subclasses of antibodies are induced. In mice, a Th1 response results in a higher production of IgG2a over IgG1 antibodies, whereas a Th2 response favors IgG1 over IgG2a[11,12]. Here, repeated intradermal administration of wtCEA DNA induced a mixed Th1/Th2 response (Fig. 6A,C). In contrast, immunization with CEA6 DNA, encoding a truncated form of CEA lacking both the N- and C-terminal signal peptides, resulted in an IgG2a/IgG1 antibody ratio of about 2, clearly shifting the immune response towards a desired Th1-type response (Fig. 6B,C). This shift towards a Th1 response resulting from intradermal immunization with an intracellular antigen could be explained by the reduced amount of soluble antigen in its native form, available for B-cell priming in the lymph node[2,37], according to the belief that antigen dose can influence the Th1/Th2 balance of an immune response[37]. This effect could be further augmented by the different glycosylation patterns of the CEA antigens since dendritic cells are more efficient in capturing and internalizing glycosylated antigens for presentation through the MHC class II pathway[38,39].

The Biojector has been used to deliver a wide range of genetically encoded antigens, e.g., HIV, rotavirus, herpes, and dengue, to both animals and human subjects, resulting in potent humoral as well as cellular immune responses[14,16,40,41,42]. However, according to our knowledge, this is the first study investigating how antigen localization might influence the induction of antibody responses after intradermal immunization with the Biojector. In conclusion, this study demonstrates that cellular localization of the DNA-encoded antigen affects both strength and quality of the humoral immune response resulting from intradermal Biojector immunization. Expression of the tumor antigen CEA as a membrane protein resulted in a mixed Th1/Th2 response with high antibody titers. Targeting of CEA to the cytoplasm reduced the antibody-stimulating capacity, but more importantly, led to an increased production of Th1-type antibody subclasses that are associated with humoral effector functions[13]. The results have important implications for design of DNA-encoded antigens intended for intradermal vaccine delivery by Biojector.

## ACKNOWLEDGMENTS

Financial support was provided by the Swedish Research Council, Cancer and Allergy Foundation, Vinnova, and The Cancer Research Funds of Radiumhemmet, Stockholm. Bioject Medical Technologies provided the Biojector device and syringes for the study. The authors do not declare any conflict of interest. Richard Stout is the Executive Vice President / Chief Medical Officer of Bioject Medical Technologies, Inc., and a shareholder of the company.
